# Olha Quem Está Voltando? Aprimorando Modelos Preditivos para Readmissão Hospitalar Pós-CRM: Insights e Perspectivas

**DOI:** 10.36660/abc.20240493

**Published:** 2024-09-17

**Authors:** Stephan Lachtermacher

**Affiliations:** 1 Unidade Cardio Intensiva Clínica Instituto Nacional de Cardiologia Rio de Janeiro RJ Brasil Instituto Nacional de Cardiologia – Unidade Cardio Intensiva Clínica, Rio de Janeiro, RJ – Brasil; 2 Unidade de Terapia Intensiva Hospital Samaritano Barra Rio de Janeiro RJ Brasil Hospital Samaritano Barra – Unidade de Terapia Intensiva, Rio de Janeiro, RJ – Brasil

**Keywords:** Modelos Teóricos, Revascularização Miocárdica, Readmissão do Paciente

O campo da cirurgia cardiovascular evolui continuamente, com a cirurgia de revascularização do miocárdio (CRM) servindo como um procedimento fundamental para o tratamento da doença arterial coronariana grave. Apesar dos avanços nas técnicas cirúrgicas e nos cuidados perioperatórios, a incidência de readmissões hospitalares dentro de 30 dias após a CRM permanece uma preocupação crítica. Estas readmissões não só têm impacto nos resultados dos pacientes, mas também sobrecarregam os recursos de saúde e aumentam os custos. Compreender os preditores de readmissão é crucial para melhorar as estratégias de cuidados pós-operatórios e otimizar os resultados dos pacientes.

O estudo intitulado " Preditores de Readmissão Hospitalar até 30 Dias de CRM em Banco de Dados Multicêntrico: Estudo de Coorte Transversal"^1^ tem como objetivo identificar variáveis associadas à readmissão precoce pós-CRM em uma população brasileira. Embora a taxa de readmissão observada tenha sido inferior à da literatura científica atual, onde as taxas variam entre 8,3% e 21,1%,^2-4^ também enfatizou causas não cardíacas. Nesse sentido, um terço de todos os pacientes readmitidos teve infecções como causa primária.^1^ Esse achado corrobora a literatura existente que destaca o impacto significativo das infecções, particularmente das infecções de sítio cirúrgico, nas taxas de readmissão.^5^ Andrade et al. sublinham a necessidade de medidas rigorosas de controlo de infecções, incluindo profilaxia antibiótica e técnicas cirúrgicas meticulosas, para mitigar estas complicações.^5,6^

Curiosamente, o estudo destacou um maior número de pacientes do sexo feminino como preditores. Embora os homens normalmente apresentem maior risco cardiovascular, até a fase da menopausa, as mulheres apresentam maior probabilidade de readmissão não planejada.^7^ Outros fatores de risco bem conhecidos incluem predisposição à anemia nos períodos pré, intra e pós-operatório e níveis mais elevados de hemoglobina glicosilada.^7^

Além desses fatores, o estudo identifica vários preditores clínicos de readmissão, incluindo comorbidades como apneia do sono e arritmias cardíacas.^1^ A apneia do sono surge como um preditor notável, associada a um risco aumentado de 11,7% de resultados adversos.^1^ Em outro estudo, Zhao et al. al. mostraram que a apneia do sono aumentou quase cinco vezes a probabilidade de readmissão não programada devido a eventos cardiovasculares, mesmo antes de inscrever pacientes para CRM,^8^ ressaltando a importância da triagem pré-operatória e do manejo dessa condição. Embora as arritmias cardíacas não tenham alcançado significância estatística neste estudo, sua relevância clínica nos cuidados pós-CRM está bem estabelecida, particularmente no manejo da fibrilação atrial.^9^

A demografia dos pacientes e os fatores de risco pré-operatórios, incluindo idade avançada e condições como diabetes, contribuem significativamente para o risco de readmissão.^10^ Variáveis intraoperatórias, como a complexidade do procedimento e o uso de circulação extracorpórea, também influenciam os resultados, com tempos operatórios mais longos e transfusão de sangue intraoperatória associada com maiores taxas de readmissão devido ao aumento das respostas inflamatórias e disfunção orgânica.^11^ O estudo corrobora esses achados e apresenta evidências sobre o uso de bombas de balão intra-aórtico, que aumentam a probabilidade de readmissão em 6,8%.^1^

Apesar desses insights, o estudo tem limitações, incluindo sua natureza retrospectiva e tamanho de amostra relativamente pequeno. A dependência de um banco de dados específico pode introduzir viés de seleção e limitar a generalização, enfatizando a necessidade de estudos prospectivos maiores para validar os resultados em populações de pacientes em todo o país, e não apenas em grandes centros como São Paulo. O aprimoramento dos modelos preditivos com variáveis adicionais, como status socioeconômico e protocolos de cuidados pós-alta, poderia refinar a estratificação de risco e melhorar a tomada de decisões clínicas.^1^

Além disso, a redução das readmissões hospitalares pós-CRM exige uma abordagem abrangente que integre determinantes clínicos, demográficos e sociais da saúde. Os hospitais devem priorizar a otimização pré-operatória, adotar práticas cirúrgicas baseadas em evidências e melhorar o monitoramento pós-operatório e os sistemas de apoio. Neste sentido, estratégias de cuidados personalizados adaptadas aos perfis individuais dos pacientes podem mitigar os riscos e melhorar os resultados globais.

Em conclusão, embora o estudo sobre os preditores de readmissão hospitalar após CABG forneça informações valiosas, os seus resultados sublinham a necessidade contínua de investigação robusta e interdisciplinar. Ao refinar modelos preditivos e implementar intervenções baseadas em evidências, os prestadores de cuidados de saúde podem melhorar o atendimento ao paciente e otimizar a utilização de recursos. A futura investigação local deve centrar-se na expansão das fontes de dados, na adoção de projetos prospectivos e na promoção de colaborações multidisciplinares para promover a medicina personalizada e melhorar os resultados pós-CRM nos países em desenvolvimento.
