# Supplementary material for: Olha Quem Está Voltando? Aprimorando Modelos Preditivos para Readmissão Hospitalar Pós-CRM: Insights e Perspectivas
Source: Arq Bras Cardiol. 2024 Sep 17;121(9):e20240493. [Article in Portuguese] doi: 10.36660/abc.20240493 (PMC11516155; doi:10.36660/abc.20240493)
Supplement: Translation

# Translation: Look Who is Coming Back? Enhancing Predictive Models for Hospital Readmission Post-CABG: Insights and Perspectives

**DOI:** 10.36660/abc.20240493i

**Authors:** Stephan Lachtermacher

**Affiliations:** 1 Rio de Janeiro RJ Brasil Instituto Nacional de Cardiologia – Unidade Cardio Intensiva Clínica, Rio de Janeiro, RJ – Brasil; 2 Rio de Janeiro RJ Brasil Hospital Samaritano Barra – Unidade de Terapia Intensiva, Rio de Janeiro, RJ – Brasil

**Keywords:** Theoretical Models, Myocardial Revascularization, Patient Readmission

The field of cardiovascular surgery continually evolves, with coronary artery bypass grafting (CABG) serving as a cornerstone procedure for managing severe coronary artery disease. Despite advancements in surgical techniques and perioperative care, the incidence of hospital readmissions within 30 days post-CABG remains a critical concern. These readmissions not only impact patient outcomes but also strain healthcare resources and increase costs. Understanding predictors of readmission is crucial for improving postoperative care strategies and optimizing patient outcomes.

The study titled " Predictors of 30-Day Hospital Readmission Following CABG in a Multicenter Database: A Cross-Sectional Study"^1^ aims to identify variables associated with early readmission post-CABG in a Brazilian population. While the observed readmission rate was lower than current scientific literature, where rates vary between 8.3% and 21.1%,^2-4^ it also emphasized non-cardiac-related causes. In this matter, one-third of all readmitted patients had infections as a primary cause.^1^ This finding corroborates existing literature highlighting the significant impact of infections, particularly surgical site infections, on readmission rates.^5^ Andrade et al. underscore the imperative for stringent infection control measures, including antibiotic prophylaxis and meticulous surgical techniques, to mitigate these complications.^5,6^

Interestingly, the study highlighted a higher number of female patients as predictors. Although men typically exhibit higher cardiovascular risk, until the menopause phase, female shows a higher likelihood of unplanned readmission.^7^ Other well-known risk factors include predisposition to anemia in the pre-, intra-, and postoperative periods and higher glycosylated hemoglobin levels.^7^

Beyond these factors, the study identifies several clinical predictors of readmission, including comorbidities such as sleep apnea and cardiac arrhythmias.^1^ Sleep apnea emerges as a notable predictor, associated with an 11.7% increased risk of adverse outcomes.^1^ In another study, Zhao et al. showed that sleep apnea increased the likelihood of unscheduled re-admission due to cardiovascular events nearly fivefold, even before enrolling patients for CABG,^8^ underscoring the importance of preoperative screening and management of this condition. While cardiac arrhythmias did not reach statistical significance in this study, their clinical relevance in post-CABG care is well-established, particularly in managing atrial fibrillation.^9^

Patient demographics and preoperative risk factors, including advanced age and conditions like diabetes, significantly contribute to readmission risk.^10^ Intraoperative variables such as procedural complexity and the use of cardiopulmonary bypass (CPB) also influence outcomes, with longer operative times and intraoperative blood transfusion associated with higher readmission rates due to increased inflammatory responses and organ dysfunction.^11^ The study corroborates these findings and introduces evidence on the use of intra-aortic balloon pumps, which increase readmission likelihood by 6.8%.^1^

Despite these insights, the study has limitations, including its retrospective nature and relatively small sample size. The reliance on a specific database may introduce selection bias and limit generalizability, emphasizing the need for larger, prospective studies to validate findings across patient populations nationwide rather than just within large centers like São Paulo. Enhancing predictive models with additional variables such as socio-economic status and post-discharge care protocols could refine risk stratification and improve clinical decision-making.^1^

Furthermore, reducing hospital readmissions post-CABG demands a comprehensive approach integrating clinical, demographic, and social determinants of health. Hospitals must prioritize preoperative optimization, adopt evidence-based surgical practices, and enhance postoperative monitoring and support systems. In this regard, personalized care strategies tailored to individual patient profiles can mitigate risks and improve overall outcomes.

In conclusion, while the study on predictors of hospital readmission following CABG provides valuable insights, its findings underscore the ongoing need for robust, interdisciplinary research. By refining predictive models and implementing evidence-based interventions, healthcare providers can enhance patient care and optimize resource utilization. Future local research should focus on expanding data sources, embracing prospective designs, and fostering multidisciplinary collaborations to advance personalized medicine and improve post-CABG outcomes in developing countries.
